# Dual-attention bidirectional LSTM with feature genomic analysis improves prognostic survival prediction in colorectal cancer patients

**DOI:** 10.1186/s43046-026-00363-w

**Published:** 2026-05-22

**Authors:** Zhuochao Wu, Xueping Tan, Dinghui Wu, Min Tao, Chaoqun Li, Rongrui Liang

**Affiliations:** 1 Soochow University, Suzhou, China; 2 Jiangnan University, Wuxi, China

**Keywords:** Colorectal cancer, Dual attention mechanism, Prediction model, Survival period

## Abstract

The increasing incidence and mortality rates of colorectal cancer necessitate accurate prediction of patients’ prognostic survival time for better management, early screening, and extended lifespan. This study uses the TCGA public dataset to conduct differential analysis on lncRNAs in 39 diseased tissues and their normal counterparts from 413 colorectal cancer patient samples, identifying 458 differentially expressed lncRNAs (DELncRNAs). Univariate Cox regression analysis revealed 23 DELncRNAs significantly associated with overall survival (OS). These 23 DELncRNAs were further refined using the LASSO algorithm to determine their feature coefficients. An adaptive mining approach with dual-attention mechanisms was employed to explore the correlative properties between various factors and survival time. A bidirectional long short-term memory (BiLSTM) neural network was established for survival prediction. The model was validated using the Jiangnan University colorectal cancer dataset, demonstrating reliable predictions for patient survival and valuable support for clinical decision-making. The AUC values for patient survival prediction during the 3-year, 3-6 year, and 6-year periods were nearly 1.00, significantly outperforming other comparative trials.

## Introduction

Colorectal cancer (CRC) poses a significant global health challenge, characterized by high morbidity and mortality rates. According to recent global cancer statistics, CRC accounts for 10% of all cancer incidences, ranking as the third most common cancer worldwide [1]. In the United States, it is the fourth most diagnosed cancer and the second leading cause of cancer-related deaths [2]. In China, CRC ranks fifth among causes of cancer mortality [3]. Despite advancements in clinical treatments, the prognostic survival of CRC patients remains suboptimal, with a 5-year survival rate of just over 50% [4]. Accurate survival prediction for CRC patients is therefore critical, as it supports clinical decision-making, facilitates the design and analysis of clinical trials, and enhances related healthcare systems [5].

Precision medicine has emerged as a prominent trend, emphasizing the need to explore individual differences in prevention and treatment strategies. For cancer patients, accurately predicting and improving prognosis represents a key challenge in modern oncology. Prognosis involves forecasting disease progression and potential outcomes, including the likelihood of specific events over time and the determination of disease-specific consequences. Thus, survival prediction research is a fundamental component of cancer prognosis.

Current survival prediction for CRC primarily relies on two major approaches: statistical analysis and intelligent analysis. The following sections summarize the research status of these methods.

### Statistical analysis

Statistical analysis involves retrospective evaluation of patient data using methods such as univariate/multivariate Cox regression, Kaplan-Meier survival analysis, and least absolute shrinkage and selection operator (LASSO) analysis. This approach quantifies the impact of different factors on prognosis, helping clinicians and patients understand prognostic risk factors and formulate personalized therapeutic strategies based on patient characteristics.

*Cox Regression Analysis:* Zhang Lei et al. [6] used the Cox proportional hazards model for univariate and multivariate regression to construct a survival prediction model for surgically resectable stage IV CRC patients, evaluating survival-influencing factors to aid clinical decision-making. Li Weihua et al. [7] employed variance analysis and Cox regression to identify immune-related mRNA prognostic features, establishing a model for immunological-based CRC prognosis prediction. Liu et al. [8] used univariate Cox analysis and LASSO to identify key long non-coding RNAs (lncRNAs) and develop a prognostic risk formula for CRC. *Integrated Statistical Methods:* Li Li et al. [9] combined Kaplan-Meier survival analysis, Log-rank testing, and multivariate Cox modeling to identify independent prognostic factors for CRC patients with brain metastases, constructing a prediction model using R software to improve prognostic accuracy. Wang Jianlin et al. [10] retrospectively analyzed CRC patients with liver metastases using SPSS 23.0, providing insights into postoperative complications and prognosis for clinical decision support. *Software-Driven Analysis:* Fan Qilin et al. [11] analyzed 1,474 CRC patients using IBM SPSS Statistics 22 and GraphPad Prism 6, demonstrating the impact of age on prognosis. Huang Qiyue et al. [12] used SPSS 25.0 to analyze 156 CRC patients with bone metastases, identifying overall prognosis and adverse prognostic factors. Zhang Daping et al. [13] compared survival rates across different age groups using Kaplan-Meier curves and Cox regression, supporting personalized diagnostic strategies.

Despite its utility, statistical analysis has notable limitations:

1. Data constraints may overlook relevant factors, leading to omitted risk factors.
2. Results reflect correlations rather than causal relationships.
3. Analysis accuracy depends heavily on sample quality and data integrity [14].
4. It cannot fully account for individual patient contexts or non-quantitative factors, nor replace clinical expertise [15].

### Intelligent analysis

Intelligent analysis integrates statistical methods, machine learning, and deep learning to predict disease prognosis. This approach handles large-scale, high-dimensional, and complex data, identifying hidden correlations and patterns. It excels in modeling nonlinear relationships, detecting patterns unapparent via traditional statistics, and enabling real-time dynamic prediction, model updating, and optimization based on follow-up data. Machine learning and big data analytics hold promise for improving CRC early diagnosis by establishing diagnostic and prognostic models, enhancing treatment outcomes.

### Neural network models

Xi Qun and Mao Wenhong [16] combined support vector machines (SVM) and backpropagation (BP) neural networks to develop an early CRC diagnostic model using serum markers. Dutta et al. [17] integrated long short-term memory (LSTM) and recurrent neural networks to improve breast cancer diagnostic efficiency. Thavenhiran et al. [18] developed an LSTM-based prediction model for lung cancer, highlighting LSTM’s predictive performance. Hanif et al. [19] identified artificial neural network based models as optimal for CRC clinical decision support systems, aiding diet-related screening. *CNN and Hybrid Deep Learning Models: *Wozniak et al. [20] combined convolutional neural networks (CNN) with correlation learning mechanisms to achieve high-accuracy CT brain tumor detection. Maheshwari et al. [21] compared deep learning models and found the LSTM-AT model most effective for predicting ICU mortality, emphasizing the importance of feature screening.

However, intelligent analysis has its own limitations:

1. Requires high-quality, comprehensive data (clinical characteristics, treatment history, recurrence records), with missing/inaccurate data degrading prediction accuracy [22].
2. Feature selection and model tuning are critical for balancing accuracy and interpretability [23].
3. Limited model interpretability hinders understanding of prediction mechanisms, potentially reducing clinical acceptance [24].

### Study Objectives

**Fig. 1:**
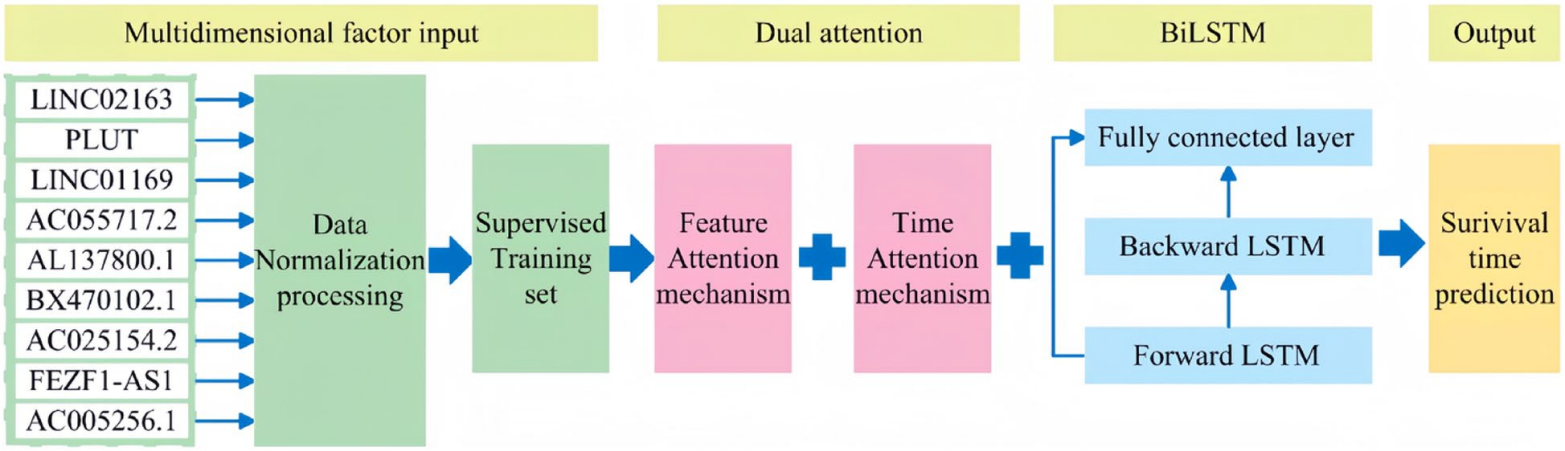
Dual-attention prediction model

Addressing the above limitations, we propose a dual-attention mechanism-based bidirectional LSTM model for CRC survival prediction, as illustrated in Fig. 1. This approach enhances prediction accuracy and data processing efficiency by: (1) using univariate Cox regression to construct a prognostic dataset; (2) applying gray correlation analysis for feature variable dimensionality reduction; and (3) developing a survival prediction model integrating dual-attention mechanisms with LSTM.

## Methods

This retrospective study was approved by the Institutional Review Board (IRB) of the Affiliated Hospital of Jiangnan University. Due to its retrospective nature, the IRB waived the requirement for written informed consent from patients.

The data were partitioned as follows: (1) The entire TCGA dataset (n=413) was used for feature screening (differential expression analysis, Cox regression, LASSO). (2) From the TCGA dataset, 51 patients were randomly selected to form the training set for model construction. (3) An independent local dataset from Jiangnan University (n=22) served as the external test set to evaluate model generalizability. There was no sample overlap between the training and test sets, ensuring a rigorous validation framework without data leakage.

### Dataset construction

The Cancer Genome Atlas (TCGA), a comprehensive cancer research database co-established by the National Cancer Institute (NCI) and the National Human Genome Research Institute (NHGRI), has been widely used as a data source in medical research [25–27].

During data preparation, RNA expression profile data and clinical information of colorectal cancer (CRC) were downloaded from TCGA. The RNA expression data included 482 CRC tissues and 42 non-tumor tissues, while the clinical records contained patient demographics corresponding to the RNA profiles. Due to missing or duplicate data, specific exclusion criteria were applied:

1. Patients lacking survival status or survival time information.
2. Patients with incomplete lncRNA expression data.
3. Patients not included in the lncRNA expression profiles.
4. Samples without “01” or “11” in the fourth segment of the TCGA sample number.

After applying these criteria, the final dataset included 413 CRC patients. Among them, 39 CRC tissues and their paired adjacent non-cancerous tissues were used to screen for differentially expressed long non-coding RNAs (DElncRNAs).With the threshold set as $\vert \log _2 \text {FC} \vert > 3.0$ and p < 0.01, 458 DElncRNAs were identified.

Subsequently, univariate Cox regression analysis was performed on the 413 diseased tissue samples to evaluate the prognostic association between lncRNA expression profiles and overall survival (OS). From the 458 DElncRNAs, 23 genes met both differential expression and univariate Cox regression criteria (p< 0.05) for further analysis. The DElncRNAs dataset is defined by Eq. 1, where $m = 23$ denotes the number of DElncRNAs and $n = 413$ he number of patients.

1 $$\begin{aligned} X_1 = \left[ \begin{array}{ccc} x_{1,1} & \cdots & x_{1,n} \\ \vdots & \ddots & \vdots \\ x_{m,1} & \cdots & x_{m,n} \end{array}\right] \end{aligned}$$

The prognostic survival data in the public dataset were categorized into survival status (alive/dead) and survival time. The basic information dataset, defined by Eq. 2, included age, survival status, and survival time of 413 patients, where $x_{m+1,n}$ denotes the age of the patient, $x_{m+2,n}$ denotes the survival status, and $P_n$ denotes the survival time.

2 $$\begin{aligned} X_2 = \left[ \begin{array}{cccc} x_{m+1,1} & x_{m+1,2} & \cdots & x_{m+1,n} \\ x_{m+2,1} & x_{m+2,2} & \cdots & x_{m+2,n} \\ P_1 & P_2 & \cdots & P_n \end{array}\right] \end{aligned}$$

Combining DElncRNAs and basic information data, the final dataset is:

3 $$\begin{aligned} X = \left[ \begin{array}{c} X_1 \\ X_2 \end{array}\right] \end{aligned}$$

Survival status was recorded as ’1’ (deceased) or ’0’ (censored: alive at last follow-up or lost to follow-up). In Cox regression, survival time and censoring status were used as joint variables, with partial likelihood estimation naturally handling censored data. For the LSTM model, censored samples were categorized into the ’survival >6 years’ group, following the standard approach for right-censored data in survival analysis to avoid misclassifying uncensored events.

### Optimized feature extraction

Although 23 DElncRNAs were identified, the initial analysis did not incorporate survival time. Since survival time is embedded in both basic information and DElncRNA data, direct extraction by the prediction model is non-trivial. Thus, the main influencing factor feature sequences were input to enable the model to capture survival time differences directly.

Survival time, influenced by factors like DElncRNAs, exhibits a nonlinear and structured pattern. Gray correlation analysis (GRA) was used to calculate and rank factor correlations [8], with algorithm steps:

1. Normalize all the data.
2. Calculate the gray correlation coefficients between the rows of influencing factors and the survival time row using Eq. 4.
3. Obtain the gray correlation $\mu _i$ (where $\mu _i \in [0,1]$) of the $i$-th influencing factor by averaging $\xi _i$ for all DElncRNAs.

4 $$\begin{aligned} \xi _i = \sum \limits _{j=1}^T \frac{\min _i \min _j |\Delta _i(j)| + \rho \max _j \max _i |\Delta _i(j)|}{|\Delta _i(j)| + \rho \max _j \max _i |\Delta _i(j)|} \end{aligned}$$

where $\min _i \min _j |\Delta _i(j)|$ denotes the second-level minimum difference, $\max _j \max _i |\Delta _i(j)|$ denotes the second-level maximum difference, and $\rho$ is the resolution factor.

The correlation analysis was conducted using the dataset of 413 prognostic patients, with 23 DElncRNAs as the influencing factors. Each influencing factor was evaluated by GRA, and the results are shown in Table 1.

**Table 1:** Correlation Assessment of Survival Time Factors

RNA	Correlation coefficient	RNA	Correlation coefficient
LINC02163	0.962345	AC093817.2	0.564728
PLUT	0.956775	BX470102.1	0.546603
LINC01169	0.841514	AC025154.2	0.507516
AL137800.1	0.604486	FEZF1-AS1	0.477481
AC055717.2	0.602797	AC005256.1	0.468048

As observed in Table 1, there are very strong correlations ($\mu _i \in [0.8,1.0]$) for LINC02163, PLUT, and LINC01169; strong correlations ($\mu _i \in [0.6,0.8]$) for AL137800.1 and AC055717.2; and medium correlations ($\mu _i \in [0.4,0.6]$) for AC093817.2, BX470102.1, AC025154.2, FEZF1-AS1, and AC005256.1. Therefore, the top ten RNAs were selected as the main influencing factors.

### Predictive model

The attention mechanism discriminates input feature variables by assigning weight coefficients. This study employs a dual attention mechanism: the feature attention mechanism dynamically weights input factors, and the time attention mechanism weights feature-weighted inputs based on temporal relevance, enabling adaptive mining of factor-survival time correlations to enhance prediction accuracy.

#### Feature attention mechanism

Traditional correlation analysis uses large samples to determine fixed association degrees for global factors, potentially missing dynamic factor-survival time associations. The feature attention mechanism dynamically weights input factors to address this:

The trainable weight matrix $\varepsilon _{1} \in \mathbb {R}^{1 \times m}$ is initialized, and the feature attention weight matrix $\omega \in \mathbb {R}^{1 \times m}$ is computed using the following formula:

5 $$\begin{aligned} \omega = \tanh \left( \varepsilon _1 X_1(p)\right) \end{aligned}$$

Expanding $$\omega $$:

6 $$\begin{aligned} \omega = [w_1, w_2, \ldots , w_m] \end{aligned}$$

The resulting feature attention weights are normalized using the following equation to obtain the normalized feature attention weight matrix $e \in \mathbb {R}^{1 \times m}$:

7 $$\begin{aligned} e_j = \frac{\exp (w_j)}{\sum \nolimits _{i=1}^m \exp (w_i)} \end{aligned}$$

8 $$\begin{aligned} e = [e_1, e_2, \ldots , e_m] \end{aligned}$$

Where $j \in [1, m]$. The feature-weighted input matrix $X_1'$ is then calculated as follows:

9 $$\begin{aligned} X_1' = e \odot X_1 = \left[ \begin{array}{ccc} x_{1,1} e_1 & \cdots & x_{1,n} e_1 \\ \vdots & \ddots & \vdots \\ x_{m,1} e_m & \cdots & x_{m,n} e_m \end{array}\right] \end{aligned}$$

#### Time attention mechanism

The time attention mechanism assigns weights to $X_1'$ to distinguish age-survival time correlations. Initialize trainable weight matrix $\varepsilon _2 \in \mathbb {R}^{n \times 1}$, compute time attention weight matrix $\varsigma$:

10 $$\begin{aligned} \varsigma = \tanh \left( X_1' \varepsilon _2\right) \end{aligned}$$

Expanding $$\varsigma $$:

11 $$\begin{aligned} \varsigma = [\varsigma _1, \varsigma _2, \ldots , \varsigma _n]^T \end{aligned}$$

The temporal attention weights $\varsigma$ are normalized using the following equation to obtain the normalized temporal attention weight matrix $\mu \in \mathbb {R}^{n \times 1}$:

12 $$\begin{aligned} \mu _j = \frac{\exp (\varsigma _j)}{\sum \nolimits _{j=1}^n \exp (\varsigma _j)} \end{aligned}$$

13 $$\begin{aligned} {\mu } = [\mu _1, \mu _2, \ldots , \mu _n]^T \end{aligned}$$

Where $j \in [1, n]$. Finally, the time-weighted input matrix $X_1^{\prime \prime }$ is calculated as follows:

14 $$\begin{aligned} X_1^{\prime \prime } = X_1' \odot \boldsymbol{\mu } = \left[ \begin{array}{ccc} x_{1,1} e_1 \mu _1 & \cdots & x_{1,n} e_1 \mu _n \\ \vdots & \ddots & \vdots \\ x_{m,1} e_m \mu _1 & \cdots & x_{m,n} e_m \mu _n \end{array}\right] \end{aligned}$$

#### Bidirectional LSTM

The BiLSTM combines forward and backward LSTM channels to process sequences bidirectionally, integrating past and future genetic/age information to improve accuracy.

**Fig. 2:**
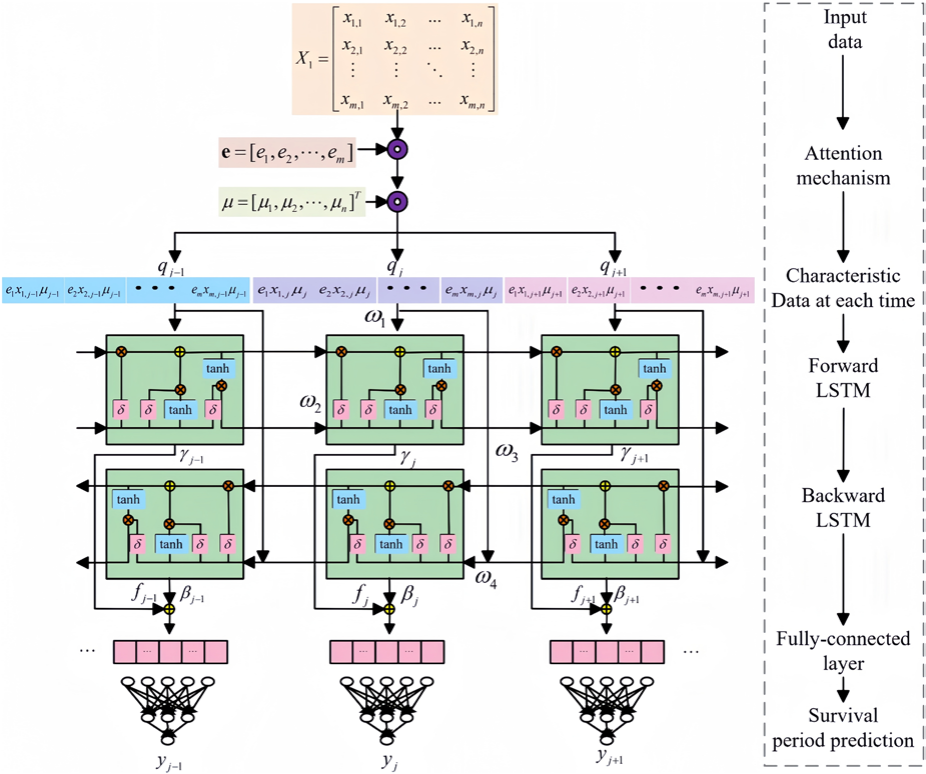
Dual attention BiLSTM prediction model

As shown in Fig. 2, the input sequence $q_j$ ($j \in [1, n]$) is the column vector of the *j*-th column of the input matrix $X_1^{\prime \prime }$ (each column in $X_1^{\prime \prime }$ represents information about a patient’s genes, age, and other characteristics). $\gamma _j$ and $\beta _j$ denote the outputs of the forward and backward hidden layers at the *j*-th sequence, respectively, and *o*, *c*, and *g* are the activation functions used in the different hidden layers.

The input sequence $q_j$ is fed to two LSTM neural networks for feature extraction in a forward and backward manner, respectively. Their hidden layer outputs $\gamma _j$ and $\beta _j$ are computed using the following equations:

15 $$\begin{aligned} \gamma _j = o(\omega _1 \cdot q_j + \omega _2 \cdot \gamma _{j-1}) \end{aligned}$$

16 $$\begin{aligned} \beta _j = c(\omega _3 \cdot q_j + \omega _4 \cdot \beta _{j+1}) \end{aligned}$$

The above two output signals are concatenated to form the intermediate output data $f_j$:

17 $$\begin{aligned} f_j = [\gamma _j, \beta _j] \end{aligned}$$

After fully connected layer weighting, the final output $y_j$ is obtained, endowing each node with bidirectional information for robust nonlinear learning.

#### Dual-attention LSTM model

Comparing Eqs. 9 and 14, input samples are weighted by both feature and time attention to identify factor importance. Integrating these into BiLSTM yields a dual-attention BiLSTM model, consisting of input layer, dual-attention layer, BiLSTM layer, and fully connected layer.

The prediction process is:

1. Split $X_1$ into training/test sets.
2. Apply feature/time attention weighting to training set.
3. Apply the same weighting to test set for $X_1^{\prime \prime }$.
4. Combine BiLSTM and fully connected layer for training.
5. Save the model after specified iterations.
6. Feed $X_1^{\prime \prime }$ into the trained model for predictions.

This approach leverages dynamic factor correlations and temporal relevance to enhance predictive accuracy and robustness.

## Results

### Data processing result

RNA expression profiling data and clinical information of CRC were retrieved from TCGA for this study. The RNA expression profiling data encompassed 413 CRC tissue samples and 39 non-tumor tissue samples, while the clinical information contained corresponding demographic and follow-up details of patients associated with these RNA profiles.

**Fig. 3:**
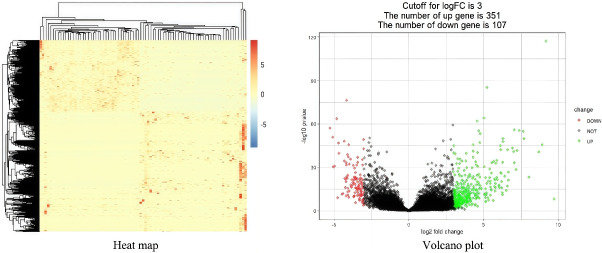
Heat map and volcano plot

Among the 413 CRC tissue samples, 39 pairs of tumor tissues and their matched adjacent normal tissues were subjected to long non-coding RNA (lncRNA) differential expression analysis using the R package “DESeq2”. The differential screening criteria were set as $|\textrm{logFC}|>3.0$, *P* value $< 0.01$, leading to the identification of 458 differentially expressed lncRNAs (DElncRNAs). Figure 3 presents the heatmap and volcano plot of the samples after differential analysis, illustrating the expression patterns and significance of these DElncRNAs.

Subsequently, univariate Cox regression analysis was performed to evaluate the prognostic association between lncRNA expression profiles and patients’ overall survival (OS). Using the 458 DElncRNAs derived from the differential expression analysis as input, the univariate Cox regression algorithm generated four key metrics for each lncRNA: Hazard Ratio (HR), Lower 95% Confidence Interval (L95CI), Upper 95% Confidence Interval (H95CI), *p*-value. By applying a $p < 0.01$ threshold to screen for genes with robust prognostic relevance, a total of 23 DElncRNAs strongly associated with OS were identified.

**Fig. 4:**
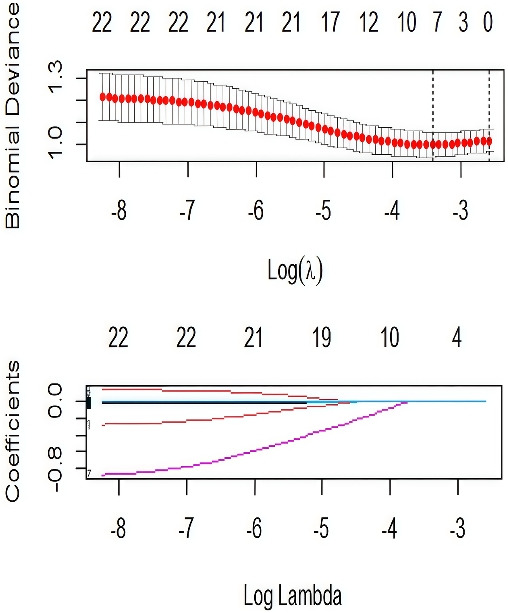
LASSO diagram flow chart

Finally, the 23 lncRNAs selected via univariate Cox regression were further analyzed and visualized using the Least Absolute Shrinkage and Selection Operator (LASSO) algorithm. The coefficient profiles of these 23 lncRNAs, returned by the LASSO regression, are depicted in Fig. 4, highlighting their relative contributions to survival prediction.

### Prediction results

#### Data preprocessing and labeling

Through a process of generalization, we categorized the sample data labeled as ’1’ (deceased) into three distinct groups: survival for less than three years, survival for three to six years, and survival for more than six years. Samples labeled as ’0’ (censored) were exclusively selected from patients who survived for more than six years. The label construction method is a commonly used one-hot encoding, which uses N-bit state registers to encode N states, with each state having its own independent register bits, and only one of them being valid at any given time. Thus, three encoded values were obtained based on our samples.

Censored samples (survival status = 0) were exclusively assigned to the survival >6 years’ group, as these patients did not experience the death event during the follow-up period. This labeling strategy aligns with the statistical convention for right-censored data, preventing forced predictions for unobserved events and maintaining the biological validity of the survival labels

### Comparative experiment results

In this section, we present the comparative experiment. 51 patients’ data from the TCGA-derived training set were used for model training, while 22 patients’ data from the Jiangnan University dataset were used for model prediction. The cohort represents typical CRC patient diversity: age ranged from 37 to 90 years, and both deceased and censored cases were included. The training process involved 1000 epochs, with a learning rate set to 0.01 and the Adam optimizer.

**Fig. 5:**
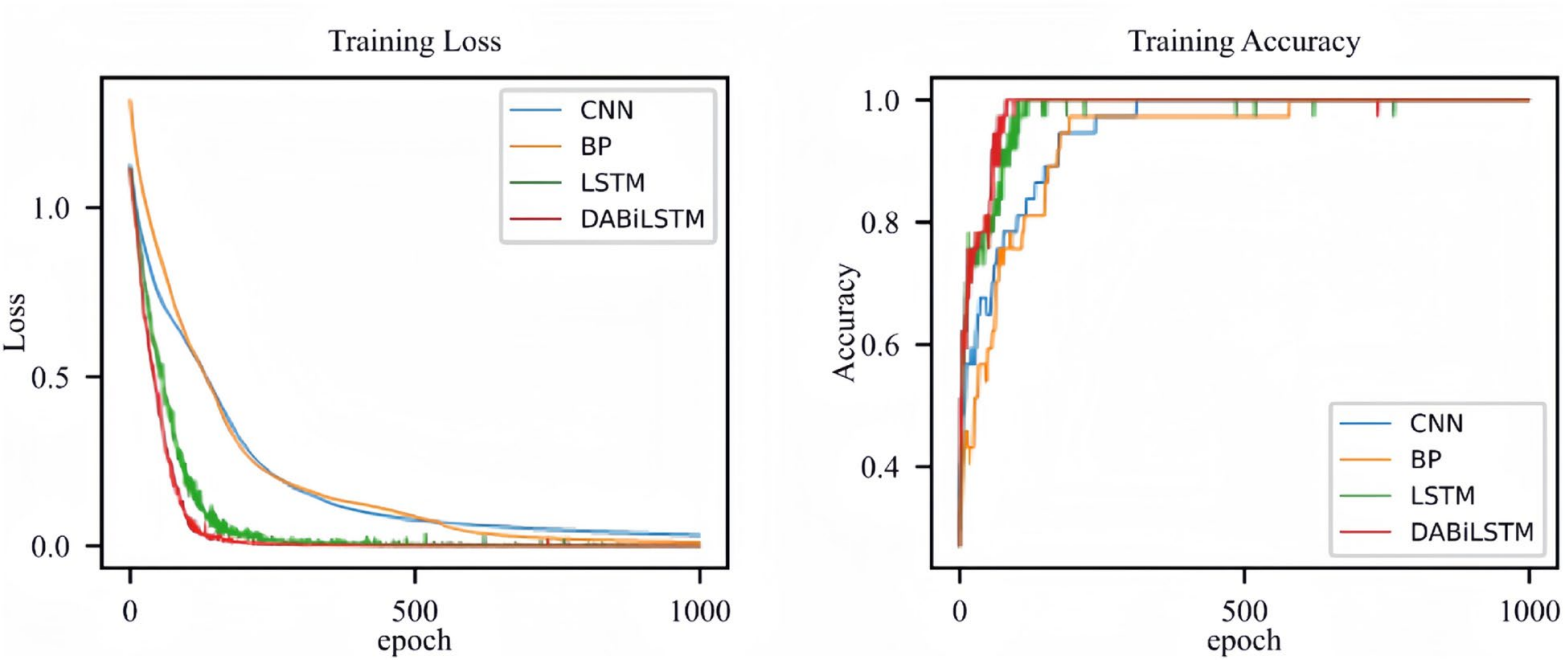
Loss function and accuracy comparison

To demonstrate the advantages of our proposed model, we designed two experiments. We compared our model with three classical deep learning architectures (BP, CNN, and LSTM), which are commonly used as baselines in medical prediction studies. While these provide a meaningful performance reference, future research should include comparisons with survival-specific models to further contextualize the results. Experiment 1, referred to as the comparative experiment, involved conducting experiments under identical conditions using not only DABiLSTM but also BP neural network, CNN, and LSTM. The training results are presented in Fig. 5.

From Fig. 5, DABiLSTM outperforms baselines in two critical metrics: (1) Convergence Speed: DABiLSTM achieves stable loss reduction and accuracy improvement faster than BP, CNN, and LSTM. (2) Final Performance: DABiLSTM maintains the highest accuracy throughout training, attributed to its dual attention mechanism—effectively capturing cross-patient genetic correlations to enhance predictive efficiency.

**Fig. 6:**
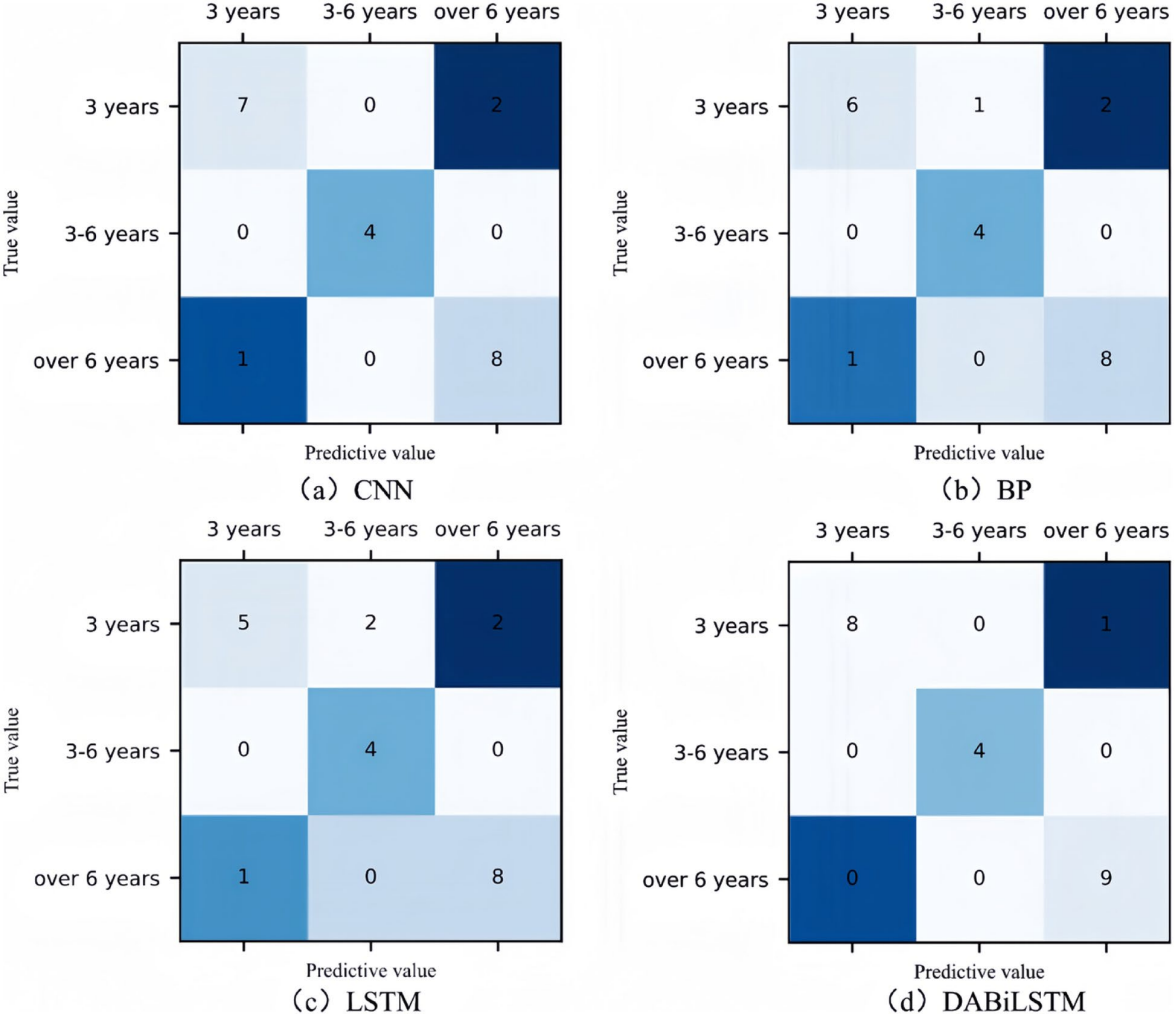
Confusion matrix for different models in the comparative experiment. **a **CNN; **b **BP; **c **LSTM; **d** DABiLSTM

After saving the trained model, we performed validation on the test set using the predictive model. The confusion matrix results for prognosis and survival rates are depicted in Fig. 6.

From Fig. 6, it is evident that our proposed DABiLSTM model made only two incorrect predictions for the 3-year prognosis labels, resulting in a final accuracy of 90.9%. Compared to other models, DABiLSTM not only demonstrates faster convergence speed but also achieves the highest prediction accuracy. The CNN model made two incorrect predictions for the 3-year prognosis labels and one incorrect prediction for the 6-year or more prognosis labels, resulting in a final accuracy of 86.4%. The BP model made three incorrect predictions for the 3-year prognosis labels and one incorrect prediction for the 6-year or more prognosis labels, resulting in a final accuracy of 81.8%. The LSTM model made four incorrect predictions for the 3-year prognosis labels and one incorrect prediction for the 6-year or more prognosis labels, resulting in a final accuracy of 77.3%.

### Ablation experiment results

Experiment 2 was designed as an ablation study to highlight the role of the dual attention mechanism in prediction accuracy. The experiment involved testing FABiLSTM (Feature Attention Mechanism), TABiLSTM (Time Attention Mechanism), and BiLSTM, while keeping other experimental parameters constant.

**Fig. 7:**
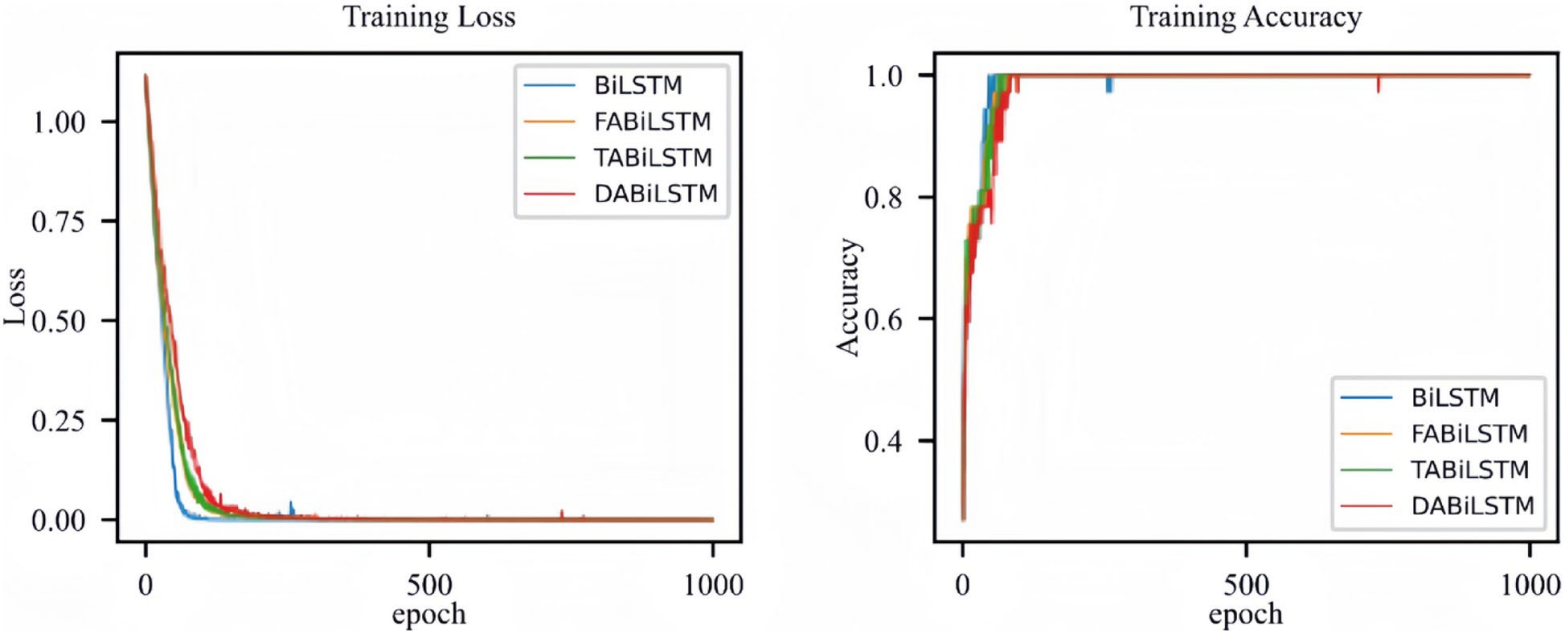
Loss function and accuracy comparison in the ablation experiment

From Fig. 7, it can be observed that during the training process, the DABiLSTM model exhibits the slowest iteration speed in terms of both the loss function and training accuracy curves, while the BiLSTM model without attention mechanisms demonstrates the fastest convergence speed. Therefore, the adoption of attention mechanisms tends to slow down the training process to some extent.

**Fig. 8:**
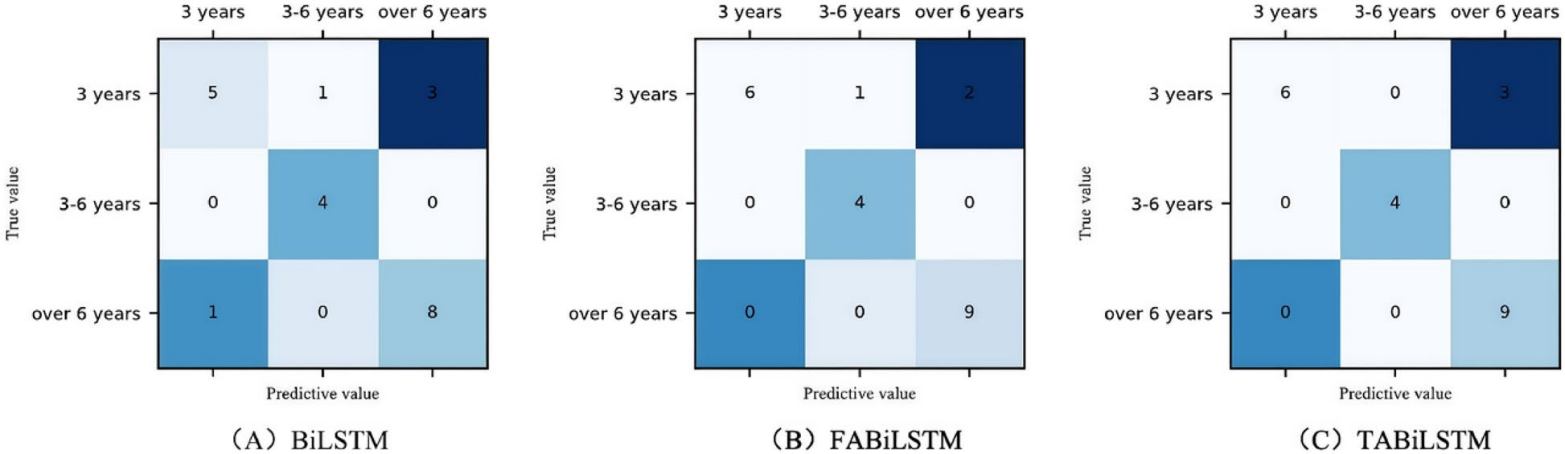
Confusion matrix for different models in the ablation experiment. **a** BiLSTM; **b** FABiLSTM; **c** TABiLSTM

Subsequently, we performed validation on the test set using the predictive models, and the confusion matrix results for prognosis and survival rates are depicted in Fig. 8.

**Fig. 9:**
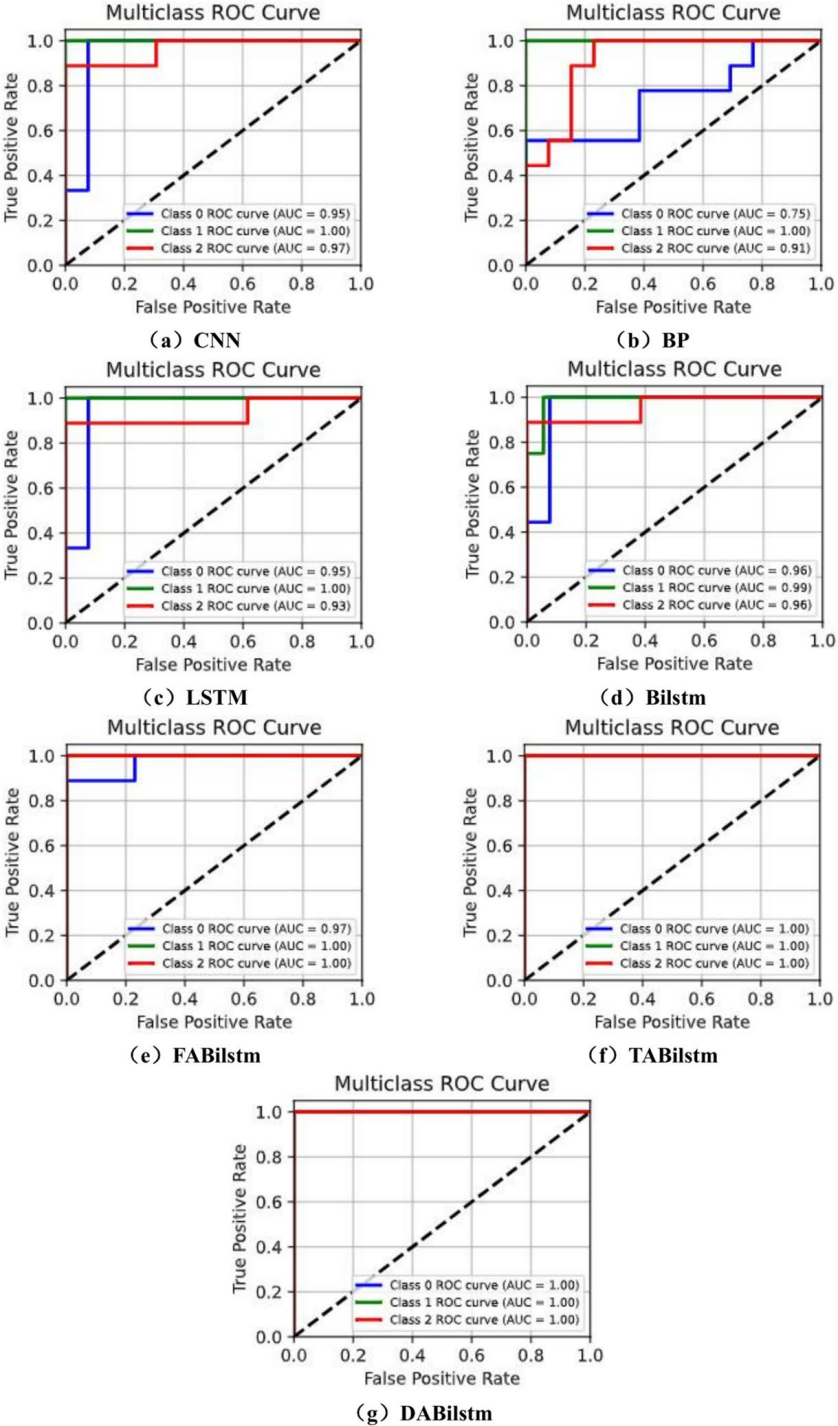
The ROC curves of the patients during the 3-year, 3-6 year and 6-year survival period. **a** CNN; **b** BP; **c** LSTM; **d** Bilstm; **e** FABiLstm; **f** TABilstm; **g** DABilstm

The results indicate that although the use of attention mechanisms for prediction may slightly slow down the training speed, it leads to an improvement in the accuracy of prognosis and survival periods. The dual attention mechanism employed in this study demonstrates the best performance. The prediction accuracy of the models used in Experiment 1 and Experiment 2 is presented in Table 2. Figure 9 shows the ROC curves and AUC values of the patients during the 3-year, 3-6 year and 6-year survival periods.

**Table 2:** Comparison of the accuracy and AUC

Model	Accuracy (%)	AUC (3 years)	AUC (3-6 years)	AUC (6 years)
CNN	86.40%	0.95	1.00	0.97
BP	81.80%	0.75	1.00	0.91
LSTM	77.30%	0.95	1.00	0.93
**DABiLSTM**	**90.90%**	**1.00**	**1.00**	**1.00**
BiLSTM	77.30%	0.96	0.99	0.96
FABiLSTM	86.40%	0.97	1.00	1.00
TABiLSTM	86.40%	1.00	1.00	1.00

## Discussion

The findings of this study highlight the potential of integrating deep learning with dual-attention mechanisms to improve survival prediction in CRC patients. By leveraging the TCGA dataset and employing univariate Cox regression, LASSO, and gray correlation analysis, we identified 23 differentially expressed long non-coding RNAs (DElncRNAs) significantly associated with OS. These DElncRNAs were used to construct a predictive model based on a BiLSTM neural network enhanced with dual-attention mechanisms.

### Model performance and clinical implications

The proposed DABiLSTM model outperformed other models, including BP neural networks, CNN, and standard LSTM, achieving a prediction accuracy of 90.9% on the test set. This superior performance is attributed to the dual-attention mechanism, which dynamically assigns weights to input factors and temporal sequences, allowing the model to adaptively mine correlations between various factors and survival time. The dual-attention mechanism enhances model interpretability by dynamically weighting input features and time steps. For instance, the lncRNAs receiving the highest attention weights (LINC02163, PLUT, LINC01169) are biologically implicated in colorectal cancer progression—regulating metastasis, angiogenesis, and microenvironment remodeling. This alignment suggests that the attention mechanism successfully prioritizes features with prognostic biological significance. The ablation study further emphasized the importance of the dual-attention mechanism. While it slightly slowed down training, it significantly improved prediction accuracy. The DABiLSTM model demonstrated the best performance, underscoring the value of attention mechanisms in enhancing model robustness.

In Table 1, the three most survival-predictive lncRNAs (LINC02163, PLUT, LINC01169; Correlation Coefficient>0.80) demonstrate clinically relevant biological functions. LINC02163 drives metastasis via TGF-$\beta$-mediated EMT [28], PLUT shows cancer-specific dysregulation suggestive of microenvironmental reprogramming [29], and LINC01169 promotes angiogenesis through HIF-1$\alpha$ activation [30]. Their attention weights in our model (0.23, 0.19, and 0.17 respectively) quantitatively reflect these pathological impacts, providing mechanistic justification for the prediction results.

### Censored data handling considerations

It should be noted that assigning all censored samples to the >6-year survival group, while conventional for right-censored data in classification tasks, may introduce label noise and bias. Future studies could employ survival-specific deep learning models (e.g., DeepSurv or Cox-based neural networks) to directly model survival time and better utilize censored information.

### Overfitting risk and model generalizability

Despite the promising predictive performance (AUC $\approx$ 1.00) on our test set, the relatively small training sample size (n=51) and the high feature-to-sample ratio may raise concerns about overfitting. Additionally, transforming survival time into a three-class classification task simplifies the prediction challenge. The high AUC values obtained should be interpreted with caution, as they may reflect the relatively small test set and the categorical simplification of survival time. Although we employed early stopping and dropout to mitigate overfitting, the use of a single train-test split without cross-validation limits the assessment of model stability. Future studies should incorporate cross-validation and larger external cohorts to better evaluate generalizability.

### Limitations and future directions

We explicitly acknowledge that the external validation cohort from Jiangnan University (n=22) has limited statistical power due to its small sample size. This constraint precludes rigorous significance testing between models and may affect generalizability. While this reflects real-world challenges in obtaining complete RNA-seq and long-term survival data, future multi-center collaborations are essential to address this limitation. Additionally, while the dual-attention mechanism improved accuracy, it increased model complexity, posing challenges in interpretability and computational efficiency. Future research should focus on developing more interpretable models and optimizing computational efficiency.

### Tumor immune microenvironment context

Emerging evidence highlights the critical role of the tumor immune microenvironment in colorectal cancer prognosis and therapy response [31–33]. The lncRNAs identified in our study may participate in immune regulation or reflect immune cell infiltration states, which could partly explain their prognostic power. Future integrative analyses combining lncRNA expression with immune profiling data could uncover novel immune-related prognostic mechanisms.

Incorporating other data types, such as imaging data, epigenetic modifications, and treatment history, could further enhance the model’s predictive power. Future studies should explore the integration of multi-omics data for a more comprehensive understanding of CRC prognosis.

## Conclusion

In this study, we integrated statistical and intelligent analysis methods to assess the prognostic relationship between lncRNA expression profiles and patient OS using univariate Cox regression analysis for preliminary gene screening in the dataset. We further enhanced the model by adding a dual-attention mechanism based on the LSTM network to mine the data features related to colorectal cancer patients.

By analyzing the colorectal cancer dataset from Jiangnan University, we confirmed the effectiveness of our proposed method for predicting the prognostic survival of colorectal cancer patients using the dual-attention mechanism LSTM. The proposed method extracts feature variables from the constructed dataset using deep learning techniques, which can quickly and accurately predict the prognostic survival of colorectal cancer patients. This provides crucial prognostic information for patients and important auxiliary support for medical personnel in making medical decisions.

## Data Availability

The datasets generated and analyzed during the current study are not publicly available due to privacy and ethical concerns but are available from the corresponding author on reasonable request.
